# New York Association for the Blind

**Published:** 1909-03

**Authors:** J. Clifton Edgar

**Affiliations:** New York


### CORRESPONDENCE.

### New York Association for the Blind.

SPECIAL COMMITTEE ON THE PREVENTION OF BLINDNESS.

*Editor Buffalo Medical Journal*:

*Sir*—In the spring of 1908, the generosity of a lady in this city rendered possible the establishment of a Special Committee on the Prevention of Blindness, as a subcommittee of the New York Association for the Blind.

The object of this committee is to ascertain the direct cause of preventable blindness and to take such measures in coöperation with the medical profession as may lead to the elimination of such causes.

The prevention of ophthalmia neonatorum is the principal aim of the members of the committee, and they seek full coöperation with the medical profession, the state and city boards of health, the state and county societies, and all organisations or persons interested in the subject.

I am sending you our first two publications, in the hope that your journal in the interests of those infants who may become "unnecessarily blind," will give the matter that publicity which will aid in reducing the criminally large percentage of needlessly blind.

J. CLIFTON EDGAR.

New York, February 4. 1909.

